# A Rare Case of Colonic Sarcoidosis Presenting as a Mass

**DOI:** 10.1155/2020/8882863

**Published:** 2020-10-10

**Authors:** Haozhe Sun, Jasbir Makker, Harish Patel, Nikhitha Mantri, Ali N. Hussain, Naeem Abbas

**Affiliations:** ^1^Department of Internal Medicine, Bronx Care Health Systems–Affiliate of Mount Sinai Hospital Systems, 1650 Grand Concourse, Bronx, NY 10457, USA; ^2^Division of Gastroenterology, Bronx Care Health Systems–Affiliate of Mount Sinai Hospital Systems, 1650 Grand Concourse, Bronx, NY 10457, USA; ^3^Department of Gastroenterology, Advantage Care Physician, 260 W Sunrise Highway Stream Valley, Rosedale, NY 11581, USA

## Abstract

**Introduction:**

Sarcoidosis is a common multisystem chronic inflammatory disease of an unidentified inciting etiology. The most common initial manifestations of this disease involve the pulmonary system, and involvement of the gastrointestinal tract is rare. Sarcoidosis of the gastrointestinal tract occurs in an oral-anal gradient, with the esophagus and stomach being the most commonly involved sites, while colonic involvement remains extremely rare. *Case Presentation*. We present a case of a 24-year-old African American man who was evaluated for persistent abdominal pain, chronic diarrhea, and weight loss. Workup for infectious etiologies and celiac disease was unrevealing. An inflammatory mass in the hepatic flexure was found during colonoscopy, and a computed tomography (CT) scan of the abdomen was significant for circumferential thickening of the cecum and ascending colon, along with nodular thickening of the peritoneum without enhancement. Malignancy and inflammatory bowel disease were the initial differentials. A peritoneal biopsy was also performed. Pathology of the colon and peritoneal biopsy was significant for the presence of noncaseating granulomas and confluent granulomatous inflammation. The patient was diagnosed with colonic sarcoidosis, and treatment with corticosteroids was initiated. Symptoms resolved with treatment, and a follow-up colonoscopy five months later showed interval healing.

**Conclusion:**

Although rare, colonic sarcoidosis should be considered as one of the differential diagnoses when evaluating a patient with chronic diarrhea and a mass on colonoscopy. Histopathology is the key to diagnosis as it distinguishes malignancy from sarcoidosis. Corticosteroids remain as an option for treating colonic sarcoidosis.

## 1. Introduction

Chronic diarrhea is defined as loose stool with increased frequency or urgency of more than four-week duration [1]. It is estimated that 5% of the population in the United States suffer from chronic diarrhea [2]. The predominant etiologies of chronic diarrhea include inflammatory bowel disease, irritable bowel syndrome, malabsorption syndrome, chronic pancreatitis, and intestinal infections. Most cases of chronic diarrhea require an extensive workup in order to reach a diagnosis. The unusual etiologies of chronic diarrhea continue to pose a diagnostic challenge [3–5]. Malignancy of the colon, while rare, can also manifest as chronic diarrhea [6].

Sarcoidosis is a chronic inflammatory granulomatous disease of young, middle-aged individuals with multisystem involvement that predominantly affects females [7]. The lung is the most common affected organ. Symptomatic gastrointestinal tract involvement is rare and occurs in less than 1% of patients with sarcoidosis [8]. If the gastrointestinal tract is involved, an oral-anal gradient is observed, and approximately 80% of such cases would involve the upper gastrointestinal tract. Therefore, involvement of the colon is rare in gastrointestinal sarcoidosis [9]. Abdominal pain is the most common presenting symptom for colonic sarcoidosis followed by diarrhea and weight loss [10].

We report a case of a 24-year-old African American male, who presented with persistent abdominal pain, diarrhea, and weight loss. Colonoscopy revealed a colonic mass concerning for malignancy. Initial imaging was significant for colonic wall thickening with possible peritoneal involvement. However, pathology results from the colonic and peritoneal biopsy were consistent with the diagnosis of colonic sarcoidosis. This patient did not have any pulmonary symptoms on presentation, and sarcoidosis was not in the array of the differentials until pathology results were obtained. The patient responded clinically to medical therapy, and resolution of colonic lesions was observed on repeat biopsy by colonoscopy.

## 2. Case Presentation

Our patient is a 24-year-old black male, who presented to us for the evaluation of persistent abdominal pain, diarrhea, and weight loss. He describes a six-month history of episodic, diffuse, and crampy abdominal pain. Each episode would last about five to ten minutes and was mild to moderate in intensity. The pain would resolve spontaneously, and there were no associated symptoms, aggravating, or alleviating factors. Three months prior to his presentation, he started experiencing loose, watery diarrhea which would occur every two to three days. He described having three to four loose bowel movements per day, with no presence of mucus or blood. He also complained of anorexia and a 10 lb weight loss over the previous three months.

A comprehensive review of systems was otherwise negative. He denied any fever, chills, or any relation of his diarrhea to food intake, and there was no history of nocturnal diarrhea. There was no recent antibiotic use and no history of recent travel or sick contacts. He denies any joint pain, skin rash, or mouth ulcers. There was no history of shortness of breath, cough, wheezing, chest pain, or urinary symptoms.

His past medical history was significant for Lyme disease in childhood and attention-deficit hyperactive disorder. There was no history of surgery in the past. He denied any history of smoking, alcohol use, or recreational drug use. There was no family history of inflammatory bowel disease (IBD), colon cancer, or any other GI malignancies.

On physical examination, the patient appeared malnourished, evident by temporal wasting. His vital signs were generally unremarkable. He weighed 134 lb (60.8 kg) which puts him at a BMI of 17.80 kg/m^2^. Examination of the gastrointestinal system was unremarkable, with a soft, nontender abdomen, with no visceromegaly. There was no scleral icterus or conjunctival pallor. The remainder of the physical exam was normal.

Results of routine hematology and biochemistry investigations including liver enzymes were all within normal limits. Celiac serology was negative, and stool workup including fecal leukocytes, stool culture, ova and parasites, giardia, and cryptosporidium were all negative. His carcinoembryonic antigen (CEA) was within normal limits.

**Figure 1:**
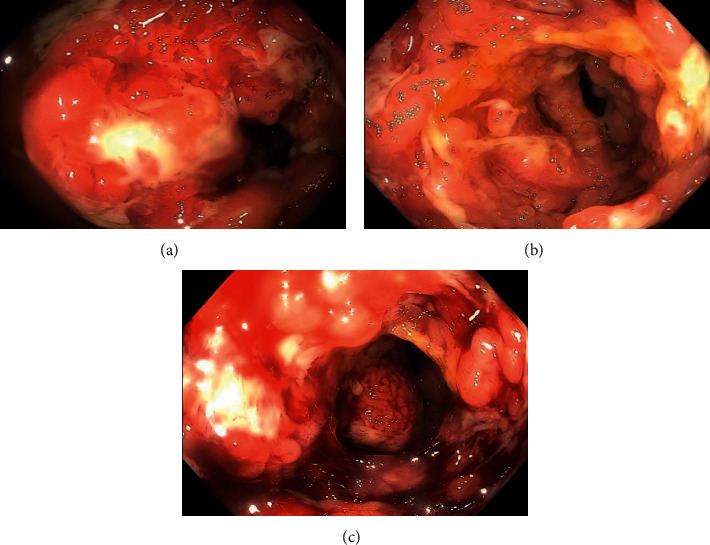
Colonoscopic findings of the hepatic flexure, demonstrating circumferential inflammatory and stenotic mass.

**Figure 2:**
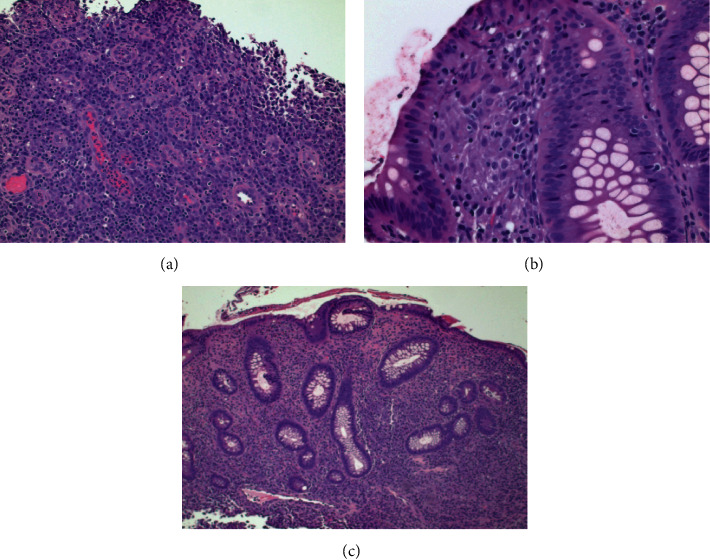
Hematoxylin and eosin (H&E) staining of the hepatic flexure mass, revealing focal active chronic colitis with ulceration, polypoid granulation tissue formation, reactive epithelial, and noncaseation granulomatous changes. Basal cell plasmacytosis could not be demonstrated. Hematoxylin and eosin (H&E) staining of the normal-appearing colon revealing the chronic colitis and preserved crypt architecture.

Colonoscopy showed a circumferentially encasing, partially obstructing, stenotic mass, with overlying granular and inflammatory mucosa in the hepatic flexure (Figures 1(a)–1(c)). The colonoscope could not be negotiated through the mass to evaluate the cecum. The colonic biopsy revealed soft consistency of the mass, and there was excess of bleeding from the biopsy site (Figure 1(c)). The colonic mucosa of the remaining part of the colon appeared normal, and random colon biopsy was obtained. The histopathology of the hepatic flexure mass was significant for focal active chronic colitis with ulceration, polypoid granulation tissue formation, reactive epithelial changes, and noncaseating granulomata formation that was consistent with granulomatous inflammation (Figure 2(a)). Special stains for tuberculosis, fungi, and other microorganisms were all negative. The crypt architecture was preserved (Figure 2(c)), and the histopathological findings were devoid of the basal cell plasmacytosis to conclude to inflammatory bowel disease. Esophagogastroduodenoscopy was performed and was unremarkable; biopsies of gastric and duodenal tissues and subsequent testing for *Helicobacter pylori* infection, celiac disease, and Whipple disease were all negative.

An abdominal CT scan of the abdomen performed with contrast was remarkable for circumferential thickening of the cecum and ascending colon without any evidence of obstruction. There were perihepatic and pelvic ascites in the cul-de-sac and nodular thickening of the peritoneum in the right lateral abdominal wall and minimally at the omentum. The patient subsequently underwent a laparoscopic peritoneal biopsy. The biopsy specimen showed noncaseating granulomas and confluent granulomatous inflammation. Special stains for tuberculosis and fungi were negative.

A CT scan of the chest was performed and revealed a slightly irregular 1 cm pulmonary nodule in the superior segment of the right lower lobe. The area of nodular sarcoid cannot be excluded. There was also minimal fissural nodularity which could represent underlying sarcoid.

**Figure 3:**
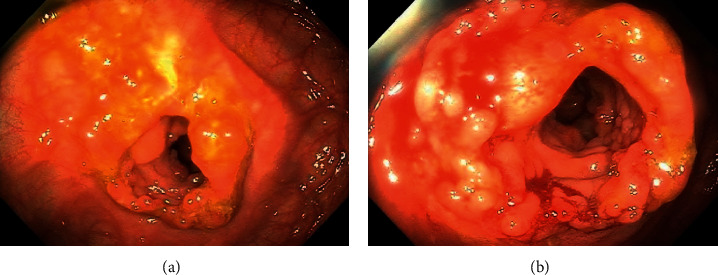
Posttreatment colonoscopy reveals interval improvement of colonic stenosis resolution of the inflammatory mass.

**Figure 4:**
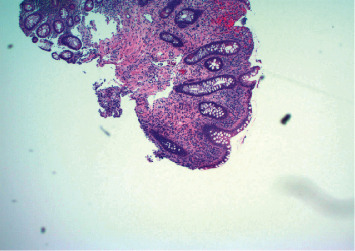
H&E staining of the colonic mucosa (poststeroid treatment) showing focal active chronic colitis without any evidence of dysplasia, metaplasia, or noncaseating granulomas.

A diagnosis of colonic sarcoidosis was made, and the patient was treated medically with oral prednisone, starting at 40 mg daily for one week with a gradual taper to a target maintenance dose of 5 mg daily over the course of eight weeks. Upon starting prednisone, the patient improved clinically with the resolution of his abdominal pain and diarrhea, along with a 20 lb weight gain, and remained in sustained symptomatic remission after three months of treatment. A colonoscopy performed five months after treatment revealed interval improvement of colonic stenosis resolution of the inflammatory mass, and the scope could be traversed through the stricture (Figure 3(a)). Some mucosal inflammation and granularity could be noted; however, there was significant improvement as compared to the index colonoscopy (Figure 3(b)). The biopsy specimens from the previously involved areas showed chronic mucosal injury with no evidence of dysplasia, malignancy, or noncaseating granulomas (Figure 4).

## 3. Discussion

Sarcoidosis presenting initially with solely gastrointestinal symptoms is extremely rare, with the current literature based on a few case reports [9]. Sarcoidosis is more prevalent in blacks with an incidence of 35.5 per 100,000 as compared to the trailing incidence of 10.9 per 100,000 in whites [11]. Women are more affected than men. However, based on the review of 31 cases of colon sarcoidosis, it is difficult to gauge ethnic or gender predominance [9].

Patients with colonic sarcoidosis often present with nonspecific signs and symptoms, such as abdominal pain, diarrhea, weight loss, and in some cases, iron deficiency anemia, hematochezia, or colonic obstruction. Combined with abdominal imaging findings of a mass, or signs of luminal obstruction, an extensive workup for a neoplastic process is often warranted.

Apart from colorectal malignancy, another important differential to consider would include the granulomatous inflammatory bowel diseases such as ulcerative colitis and particularly Crohn's disease. Both sarcoidosis and inflammatory bowel disease can share similarities in terms of extraintestinal manifestations such as erythema nodosum, arthropathy, and uveitis. Other differentials would also include colonic granulomatous disorders, hypersensitivity to foreign antigens, or infectious etiologies such as tuberculosis, syphilis, and fungal infections. It is also important to consider that these diseases could be primarily responsible for the clinical manifestations, and the coexistence of sarcoidosis is just an incidental finding.

The diagnosis of colonic sarcoidosis remains challenging, and the demonstration of noncaseating granulomas on biopsy is necessary. The resolution of granulomatous inflammation and significant clinical improvement with corticosteroid therapy point to a diagnosis of sarcoidosis in our reported case. However, in equivocal cases, clinicians should be prudent in excluding other alternative diagnoses. In such cases, diligent follow-up and re-evaluation of the patient could prove to be a sensible strategy.

The therapeutic approach to colonic sarcoidosis would usually involve surgery due to the lack of a definitive diagnosis by endoscopic biopsy or a concern for a neoplastic process. There are a few reports of responsiveness to corticosteroid therapy in gastrointestinal sarcoidosis and one with methotrexate [12]. There is no consensus with regard to the appropriate follow-up period of colonic sarcoidosis; however, the resolution of colonic masses with steroids in our case is reassuring and rules out the possibility of an alternative diagnosis.

## 4. Conclusion

Colonic involvement is an extremely rare initial presentation of sarcoidosis. Colonic sarcoidosis may present with colonic masses with peritoneal lesions and require a tissue diagnosis to rule out the alternative diagnosis of malignancy. Corticosteroids are a viable therapeutic option, and based on our current case report, surveillance is suggested to interval resolution.
